# Clinical characteristics and quality of life for rhegmatogenous retinal detachment in a tertiary centre in Ghana: a case control study

**DOI:** 10.1186/s41687-025-00952-8

**Published:** 2025-10-07

**Authors:** Akwasi Ahmed, Emmanuel Appiagyei, Jessica Sedhom, Arthur Brant, Andrew DesLauriers, Alec Bernard, Amos Aikins, Seth Lartey, Kwesi Amissah-Arthur

**Affiliations:** 1 Eye Centre, Komfo Anokye Teaching Hospital, Kumasi, Ghana; 2 Department of Optometry and Visual Science, Kwame Nkrumah University of Science and Technology, Kumasi, Ghana; 3 Tulane University School of Medicine, New Orleans, LA USA; 4 Byers Eye Institute, Stanford University, Palo Alto, CA USA; 5 University of Vermont School of Medicine, Burlington, VT USA; 6 Eye Unit, Department of Surgery, Korle Bu Teaching Hospital, Accra, Ghana; 7 Ophthalmology Unit, Department of Surgery, University of Ghana Medical School, Accra, Ghana

**Keywords:** Rhegmatogenous retinal detachment, Quality of life, Treatment delay, Sub-Saharan Africa, Primary care, Public health, Global burden of disease, Irreversible blindness

## Abstract

**Purpose:**

To assess the Quality of Life (QoL) and understand the clinical characteristics of patients in Ghana presenting with RRD compared to age-matched controls.

**Methods:**

This prospective case-control study included patients presenting with RRD at a tertiary hospital in Ghana between July 2021 and June 2022 compared to age-matched unaffected individuals. QoL was evaluated using the National Eye Institute Visual Function Questionnaire (NEI-VFQ-25). Demographic and clinical characteristics were analysed.

**Results:**

Among 180 participants (57 RRD cases, 123 controls), QoL scores ranged 50–70 for RRD patients and 60–90 for controls. The overall composite score of NEI-VFQ-25 in the RRD patients (mean 61; SD 12) was significantly lower than that of controls (mean 78; SD 15), including 8 of 12 QoL subscales (*p* < 0.001). The lowest subscale scores for RRD cases were vision-specific role difficulties (mean 48; SD 8). Floaters were the most common presenting symptom among RRD cases (75.4% vs. 1.6%) (*p* < 0.001). RDD patients presented to a primary eye centre after an average 30.8 days (SD = 70.0) and to a tertiary centre after 200.4 days (SD = 306.8).

**Conclusion:**

There is a significant reduction in the QoL for RRD patients. Early diagnosis and management of RRD are essential to mitigate the adverse impact on QoL and prevent further complications.

## Introduction

Globally, an estimated 42 million individuals are blind, with 90% of those living in Low and Middle-Income Countries (LMICs). Retinal detachment and other retinal disorders are responsible for at least 7% of blindness worldwide; however, the true prevalence may be much higher, as large portions do not have access to retinal care [1]. In LMICs, retinal disorders are often given less priority due to limited resources and a large backlog of untreated cataract and refractive error. Unlike cataract blindness, however, many retinal causes of blindness are irreversible [2].

Retinal detachment is a condition characterised by the separation of the neurosensory retina from the underlying retinal pigment epithelium (RPE), which compromises blood flow and nutrition to the photoreceptor cells that are crucial for visual perception [3]. Left untreated, this detachment results in tissue necrosis and subsequent irreversible vision loss in all patients [3]. Rhegmatogenous retinal detachment (RRD) is the most prevalent type of retinal detachment. The most common mechanism by which RRD occurs is through liquefaction of the vitreous humour exerting traction on the retina, resulting in a retina break. Fluid then enters through this break into the subretinal space and subsequently separates the neurosensory retina from the underlying RPE [4]. RRD disrupts the metabolic support between the retina and the RPE, triggering oxidative stress, inflammation, and photoreceptor cell death [4]. Key molecular changes include the release of cytokines, activation of apoptotic pathways, and glial cell responses. These events accelerate retinal degeneration, highlighting the need for timely surgical repair to prevent irreversible vision loss [3, 4].

Early identification of retinal breaks allows for prophylactic laser treatment, which is non-invasive and highly effective at preventing fluid from entering the break [5]. For patients presenting after the retina has detached, several surgical options are available, including pars plana vitrectomy (PPV), scleral buckle surgery, and pneumatic retinopexy. These procedures aim to reattach the retina and restore retinal anatomy, with high anatomical success rates when performed in a timely manner [5, 6].

In several LMICs, there is a paucity of data on the clinical characteristics and risk factors of RRD, which is thought to be attributable to a lack of awareness, delayed diagnosis, and the scarcity of specialised facilities. Patients with RRD commonly present late, and a considerable number have chronic and acute complications that, in high-income countries, are often avoided with prompt treatment [7, 8]. As the healthcare systems in Ghana and other LMICs evolve with increasing national wealth, the demand for equitable access to specialized ophthalmic care grows. However, evidence-based policies guiding the allocation of resources for retinal disease management are lacking. To address this gap, our study provides the first quantitative assessment of visual quality of life (QOL) in patients with RRD in Ghana, offering region-specific insights into the burden of this condition.

In this study, we measured QoL in patients with and without RRD presenting to the Komfo Anokye Teaching Hospital Eye Centre (KATH) to better understand the clinical characteristics and risk factors of RRD. Empiric evidence that measures QoL in these patients will help guide the development of a robust healthcare system capable of delivering timely, sight-saving treatment for all patients with RRD.

## Methods

This prospective case-control study was conducted at the Retina clinic of the Komfo Anokye Teaching Hospital (KATH), a tertiary centre in Ghana. The study included patients who were 18 years and over with RRD between July 2021 and June 2022 presenting to the retina clinic of KATH. Exclusion criteria included (1) patients with any media opacity obscuring the visualisation of the posterior segment; (2) RRD associated with vasoproliferative disorders such as proliferative diabetic retinopathy, sickle cell retinopathy or retinopathy of prematurity; (3) RRD associated with inflammatory eye disease including (cytomegalovirus retinitis, acute retinal necrosis, pars planitis, endophthalmitis) and (4) retinal re-detachment.

### Control recruitment

We employed age-matching to reduce potential confounding due to age-related differences in visual function and quality of life. Controls were recruited from patients attending the general eye clinic for routine check-ups and were matched to cases within a ± 5 year age range. Controls included patients without ocular structural and functional changes confirmed by clinical examinations and with refractive error (equivalent sphere) of up to + 3 or -3 dioptres.

### Sample size

Assuming a medium effect size (Cohen’s d = 0.5), a power of 80% (β = 0.2), and a significance level of 5% (α = 0.05), we determined that a minimum sample size of 180 participants was required (57 cases and 123 controls). All cases and controls fulfilling the inclusion criteria were consecutively included in the study until the sample size was achieved.

### Examination of cases and controls

At presentation, patients’ demographic information was documented using a structured questionnaire. The duration between symptom onset and presentation at various levels of care (primary care facility, tertiary eye centre, and retina specialist) was calculated based on patient self-report. These self-reported dates were then compared with the documented dates of first presentation at each level of the healthcare system. An assigned ophthalmic nurse measured the participant’s visual acuity using a Snellen chart, with the participant seated 6 m away from the chart. An assigned Optometrist determined the type of refractive error using objective refraction with a Humphrey^®^ Autorefractor (serial number 599–7788, manufactured by Carl Zeiss Meditec, Germany) and performed subjective refraction to ascertain the best corrected visual acuity (BCVA) for analysis. The pupils were dilated with a mixture of Phenylephrine 2.5% and Tropicamide 1% eye drops to ensure good visualisation of the fundus. A retina-trained ophthalmologist (AA) examined the anterior segment and posterior segment of each eye for cases and controls. The anterior segment was examined using the Slit Lamp (Topcon Corporation, Japan). The posterior segment was examined using a + 90 Dioptre Volk^®^ Fundus Lens with the Slit Lamp (Topcon Corporation, Japan) using slit beam illumination and by indirect ophthalmoscopy using a Heine Indirect Ophthalmoscope and a + 20 Dioptre Volk^®^ Fundus Lens with the aid of a scleral depressor to examine the peripheral retina. The presence of vitreous haemorrhage, status of macula (on/off) and extent of detachment were recorded. The diagnosis was based on clinical identification of a full-thickness retinal break associated with subretinal fluid extending from the break. For controls, the vitreous, macula, vessels, periphery, and optic disc were examined.

### Questionnaire

The NEI VFQ-25 was used to assess the quality of life of the study participants. In order to discern between general QoL and vision-specific QoL, the National Eye Institute (NEI) funded the development of a standardized questionnaire, which was ultimately modified to become the National Eye Institute Visual Function Questionnaire (NEI VFQ)-25. The copyright of the NEI VFQ-25 is owned by the National Eye Institute [9]. This-25 item survey covers the following subscales: general health (GH), general vision (GV), ocular pain (OP); difficulty with near-vision activities (NV), difficulty with distance-vision activities (DV); vision-related limitation of social functioning (SF); vision-related mental health problems (MH, 4 items), vision-related role limitations (RL); vision-related dependency on others (DP); driving difficulties (DR); difficulty with color vision (CV) and peripheral vision (PV). Each subscale is between 0 and 100. All subscales (except GH) are averaged to produce a composite score with a higher number indicative of higher QoL [9]. The questions were administered by (AA) to all participants (cases and controls) after clinical examination. Although the NEI VFQ-25 was not formally translated into the local Ghanaian language, it was administered in English, and study personnel provided real-time explanations in Twi when required. Despite this, internal consistency of the questionnaire responses was high, with a Cronbach’s alpha of 0.89, indicating that participants consistently understood and responded reliably to the items. Responses were recorded based on the predefined Likert-type scales provided in the NEI-VFQ-25 questionnaire.

### Analysis

Data was managed in Microsoft Excel and transferred to Python 3.7 on a secure laptop for analysis. Visual acuity, initially measured with Snellen chart, was converted to LogMAR at the patient level for analysis. The LogMAR (logarithm of the minimum angle of resolution) scale, quantifies visual performance by calculating the logarithmic transformation of the smallest resolvable visual angle. In this scale, lower LogMAR values correspond to better visual acuity, while higher values indicate worse acuity. The demographic and clinical characteristics of study eyes were analysed using Mann-Whitney U test and chi-square test depending on the variable of interest.

NEI VFQ-25 data was analysed using the standard method of summing Likert-type scores and calculating an overall mean. Each scale consisted of a minimum of one and maximum of four items. The standard algorithm as described by Mangione et al. [9, 10] was used to calculate the scale scores, which have a possible range of zero to 100. The higher the score, the better the vision related QOL pertinent to that specific symptom or activity. The overall composite score for the NEI-VFQ-25 was calculated by averaging the subscale scores, excluding the general health rating question. This method ensured that each subscale is given equal weight, regardless of the number of items in each subscale. Normality of the data was assessed using the Shapiro-Wilk test and found to be non-normally distributed. Consequently, the non-parametric Mann-Whitney U test was used for comparisons between groups. The level of significance for all statistical tests conducted in this study was set at *p* < 0.05.

### Ethical considerations

This study was conducted in accordance with the principles of the Declaration of Helsinki. Ethical approval was obtained from the Komfo Anokye Teaching Hospital Institutional Review Board (Reference Number: KATH IRB/AP/046/21). Written informed consent was obtained from all participants prior to their inclusion in the study. Participants were assured of the confidentiality and anonymity of their responses, and data were handled in compliance with relevant ethical and legal standards.

## Results

Overall, 180 participants were included in the study with 57 cases and 123 controls. Mean age was 48.7 ± 12.1 years in the RRD cases and 48.9 ± 16.3 years in the control group. Among the RRD cases, 35 (61.4%) were male and 22 (38.6%) were female. Educational and insurance status were not significantly different between case and control group. Demographic information is summarized in Table 1.

**Table 1:** Demographic characteristics of cases (RRD patients) and controls

Characteristics	Cases (*n* = 57)	Controls (*n* = 123)	*P* values	
Age, mean years ± SD	48.7 ± 12.1	48.9 ± 16.3		0.959^†^
Sex, n (%)				0.017*
Male	35(61.4%)	52(42.3%)		
Female	22(38.6%)	71(57.7%)		
Education, n (%)				0.756*
Tertiary	19(33.3%)	33(26.8%)		
High School	28(49.12%)	66(53.7%)		
Primary	4(7.0%)	10(8.1%)		
None	6(10.5%)	44(35.8%)		
Insurance Type, n (%)				
National Health Insurance	57 (100%)	110 (89.4%)		
Private Health Insurance	0 (0%)	13 (10.6%)		

RRD; Rhegmatogenous retinal detachment, SD; Standard Deviation

*Pearson chi-square test

† Mann-Whitney U test

At the time of presentation, mean BCVA LogMAR score was 1.77 ± 0.84 for cases and 0.44 ± 0.66 for controls. In eyes with RRD, 45 (77.6%) of cases had a BCVA of 20/200 or worse compared to only 16 (13.0%) right eye controls. Inversely, 19 (32.8%) of RRD cases compared to 87 (70.7%) of control participants had a BCVA of 20/40 or better in the better-seeing eye. Bilateral visual impairment, defined as BCVA between 20/40 and 20/200 in the better seeing eye, was present in 28 (48.3%) of RRD cases and 27 (21.9%) of controls. Bilateral blindness, defined as 20/200 or worse in the better-seeing eye, was present in 10 (17.2%) RRD cases and 9 (7.3%) controls.

Among the RRD cases, vitreous haemorrhage was present in 5 (8.8%) eyes. Thirty-five (61.4%) of cases had total retinal detachment and macula-off detachments were found in 44 (77.2%) of RRD cases. The most common presenting complaint was floaters in 43 cases (75.4%, *p* < 0.001) followed by flashes in 22 cases (37.9%, *p* < 0.001) (Table 2).

**Table 2:** Risk factors and clinical characteristics of cases (RRD patients) and controls

Characteristics	Cases (*n* = 57)	Controls (*n* = 123)	*P* values
BCVA (LogMAR), mean ± SD	1.77 ± 0.84	0.44 ± 0.66	< 0.001^†^
Refractive Status, n (%)			< 0.001*
Myopia	19(33.3%)	37(30.1%)	
Emmetropia	38(66.7%)	59(48.0%)	
Hyperopia		16(13.0%)	
Astigmatism		11(8.9%)	
Vitreous Haemorrhage, n (%)			
Present	5(8.8%)		
Absent	52(91.2%)		
Extent of retinal detachment, n (%)			
Total	35(61.4%)		
Subtotal	22(38.6%)		
Macula Status, n (%)			
Macula-on	13(22.8%)		< 0.001*
Macula-off	44(77.2%)		< 0.001*
Presenting Complaints			
Floaters	43 (75.4%)	2 (1.6%)	< 0.001*
Field Loss	20 (34.5%)	0 (0.0%)	< 0.001*
Flashers	22 (37.9%)	0 (0.0%)	< 0.001*
Falling Curtain	10 (17.2%)	0 (0.0%)	< 0.001*
Vision Loss	10 (17.2%)	48 (39.0%)	0.007*
Pain	0 (0.0%)	28 (22.8%)	< 0.001*

BCVA; Best Corrected Visual Acuity, RRD; Rhegmatogenous retinal detachment, SD; Standard Deviation

*Pearson chi-square test

† Mann-Whitney U test

The average duration between RRD symptoms and visiting a primary care facility was 30.8 days (SD = 70, *p* < 0.05), tertiary eye facility was 200.4 days (SD = 306.8, *p* < 0.05), and retina specialist was 266.77 days (SD = 438.11, *p* < 0.05). Majority of the RRD cases were referred by optometrists (*n* = 44, 77.2%), followed by ophthalmologists (*n* = 10, 17.5%) and then ophthalmic nurses at (*n* = 2, 3.5%). The mean duration of referral from optometrists to the tertiary eye centre was 193.1 ± 315.1 days, from ophthalmologists; 148.1 ± 184.7 days, and from ophthalmic nurses 33.5 ± 37.4 days. Macula-on RRD cases took a period of 21 ± 22.8 days and 223.4 ± 313.8 days for macula-off RRD to be referred to a tertiary hospital.

Almost all sub-scales and overall composite score of VFQ-25 in the RRD patients were significantly lower than that in the controls except for ocular pain, VS mental health, general vision, and colour vision. The lowest subscale scores for RRD cases were VS Role difficulties (48 ± 8) followed by general vision (51 ± 9) (Table 3).

**Table 3:** Comparison of NEI-VFQ-25 mean sub-scale scores between cases (RRD patients) and controls

Subscale (no. of questions in each subscale)	Cases Mean(± SD)	Controls Mean (± SD)	*P* value
Composite Score	61 ± 12	78 ± 15	< 0.001
Ocular pain (2)	60 ± 11	60 ± 12	0.551
VS Role difficulties (2)	48 ± 8	65 ± 23	< 0.001
VS Mental health (4)	66 ± 11	68 ± 14	0.401
General vision (1)	51 ± 9	56 ± 12	0.341
Near activities (3)	60 ± 16	70 ± 13	< 0.001
Distance activities (3)	65 ± 10	78 ± 8	< 0.001
Driving (3)	61 ± 12	80 ± 12	< 0.001
VS Dependency (3)	55 ± 16	85 ± 12	< 0.001
Peripheral vision (1)	72 ± 12	90 ± 12	< 0.001
VS Social functioning (2)	69 ± 15	75 ± 11	< 0.001
Colour vision (1)	70 ± 9	75 ± 17	0.201
General Health (1)	52 ± 12	70 ± 12	< 0.001

NEI-VFQ-25; National Eye Institute Visual Function Questionnaire

Subscales are all scored on 0-100 possible range (where 100 represents the best quality of life)

SD; Standard Deviation

VS; vision specific

*40 participants did not drive for reasons which are not vision related

Statistical significance tested using the Mann-Whitney U Test

Subgroup analysis showed that macula-on patients had a slightly higher mean score (67 ± 11) compared to macula-off patients (62 ± 17), though this difference was not statistically significant. Similarly, there were no significant differences in visual function scores between patients with total vs. subtotal retinal detachment, male vs. female, or presence vs. absence of vitreous haemorrhage (Table 4).

**Table 4:** Subgroup analysis of visual function scores of RRD patients

Parameter	Mean ± SD	Median (25% − 75%)	Min - Max
Macula			
On	67 **±** 11	68 (58–72)	53–90
Off	62 **±** 17	66 (50–74)	14–91
Severity			
Total	61 **±** 18	65 (49–73)	14–91
Subtotal	60 **±** 9	60 (55–67)	45–73
Sex			
Male	66 **±** 16	67 (58–76)	14–91
Female	60 **±** 16	64 (53–71)	26–86
VH			
Present	57 **±** 12	58 (45–63)	42–76
Absent	64 **±** 16	67 (56–73)	14–91

RRD; Rhegmatogenous Retinal Detachment, VH; Vitreous Haemorrhage

SD; Standard Deviation

Min-Max; Minimum - Maximum

## Discussion

In this study, we investigated the vision-related quality of life in patients with and without RRD in Ghana. Our findings revealed that patients with RRD in Ghana had significantly lower vision-related QoL compared to aged-matched controls. Furthermore, patients with RRD had significantly worse visual acuity and scored significantly lower on the vision-related quality of life composite score. Among the subscales analysis, social functioning, driving, near and distance activities, and VS role were all significantly impaired among RRD patients. QoL related to ocular pain, mental health, colour vision, and general vision showed no difference when compared to age-matched controls. Analysis of demographic characteristics indicate that QoL is poor in patients with RRD regardless of sex, age, occupation, insurance, or educational status. Although our inclusion and exclusion criteria ensured that RRD cases did not have other coexisting ocular pathologies, sub-analysis of visual acuity revealed that a substantial proportion of RRD patients presented with sub-optimal vision, bilateral visual impairment or blindness at the time of assessment. Only 32.8% of RRD cases had a BCVA of 20/40 or better in their better-seeing eye, compared to 70.7% in the control group. This suggests that the reduced quality of life observed among RRD patients may be primarily driven by the degree of functional visual loss; especially in both eyes, rather than by the presence of other ocular diseases. Therefore, the impact of RRD on vision-related quality of life appears to be closely linked to the extent of binocular visual acuity compromise, reinforcing the importance of early detection and management to preserve vision and maintain QoL.

A similar study in China used the Chinese version of the VFQ-25 to compare patients with RRD to healthy controls selected from the general population [10]. The RRD group in the China study had very similar composite score to the Ghana study, with a composite score of 61 in both studies. A notable difference between the RRD patients in each study was that the Ghanaian cohort scored 16 points higher on the mental health subscale. This might stem from variation in translation or cultural and linguistic variations affecting the interpretation. Additionally, it could be reflective of different attitudes towards mental health, particularly in the setting of retinal disease. In Ghana, there is generally a lack of open discussion regarding mental health issues; however, there is a strong social support network, in large part due to a culture of communalism and caretaking.

It is important to note that most studies assessing QoL in RRD patients using the NEI VFQ-25 have been conducted after surgical intervention, focusing on the recovery of visual function and the long-term impact of the procedure on patients’ quality of life. In contrast, our study assessed QoL before surgical repair, offering a unique snapshot of the burden of RRD at presentation. This distinction is critical, as our findings reflect the acute functional and psychological impact of RRD prior to any visual recovery, potentially explaining why our scores may appear lower compared to postoperative cohorts [11–17].

The Minimally Clinically Important Difference (MCID) represents the smallest change in a patient-reported outcome measure that is perceived as beneficial and would warrant a change in clinical management. For the NEI VFQ-25, the MCID has been estimated to range between 4 and 6 points for the composite score based on anchor and distribution based methods in ophthalmic populations [18]. In our study, the composite score was 61 ± 12 among RRD patients compared to 78 ± 15 in controls. This 17-point difference substantially exceeds the MCID threshold, signifying a clinically meaningful reduction in vision-related functioning. Notably, patients with macula-off detachments and those with vitreous haemorrhage exhibited even lower scores, demonstrating the compounded functional burden of anatomical severity. These findings suggest that RRD significantly impairs daily visual activities.

The significantly low QoL among patients with RRD can be addressed through early identification and treatment. The results of this study indicate that RRD patients presented to a primary eye facility on average after 1 month and only saw a retina specialist (the physician capable of offering surgery for RRD) after nearly 266 days. Most concerning is that the macula-on patients took a mean of 3 weeks to see a retinal specialist. In high-income countries, macula-on retinal detachments are typically operated on within 24 h and are considered a surgical emergency. It is difficult to discern precisely the percentage of macula-on RRDs that may have progressed to macula-off given the delay in presentation. The visual prognosis are drastically different between macula on RDs (where a patient is expected to maintain baseline visual acuity) and macula-off (where prognosis are guarded). Further studies should focus on the potential reasons for this delay in treatment culturally and economically specific to the Ghanaian population. A study from the Netherlands identified patient ignorance as the primary cause of delay in treatment wherein individuals did not identify the symptoms of RRD as urgent [19]. Notably, this delay in care was an average of 9 days. Delayed presentation in the Ghanaian population may be attributed at least in part to a lack of access to eye care facilities and specialists. At the time of writing, there are approximately eight retina specialists in Ghana, or one retina specialist per 4.69 million Ghanaian residents [20]. Furthermore, these retinal specialists are concentrated in two major cities in Ghana: Accra and Kumasi, leaving large areas without access to retinal care. For comparison, in the US there is one retina specialist for every 128,000 people [21]. This contrast underscores the dire need for increasing the number of training opportunities for retina specialists in Ghana. The lack of trained specialists results in decreased or delayed treatment of RRD and, by proxy, poor QoL for those presenting with this condition.

Given that floaters were the most common presenting symptom of RRD, this study emphasizes the importance of educating primary clinic physicians, optometrists, nurses, physician assistants and ancillary healthcare personnel on the importance of immediate referral in the setting of floaters. In Ghana, where resources are limited, it is essential to make these protocols standard of care when triaging ophthalmic patients. In this study, most referrals for RRD management came directly from an optometrist (77.2%); however, patients presentation to a tertiary eye care center was delayed an average of 193.1 days when referred by an optometrist. Future studies should investigate this delay-in-treatment in order to optimize referral networks. Residency training should ensure Ghanaian ophthalmologists and optometrists can identify RRDs through indirect exam or imaging. Scleral depression is rarely performed in general clinic; however, it is an essential skill to identify retinal tears early and treat prophylactically prior to retinal detachment.

There are several limitations to this study. While the NEI VFQ-25 is a widely used instrument for assessing the impact of visual impairment on daily activities, it primarily focuses on visual functioning and socio-emotional wellbeing rather than a comprehensive evaluation of overall quality of life. General QoL measures, such as the SF-36 or WHOQOL-BREF, include broader aspects of physical health, mental health, and social functioning, which the NEI VFQ-25 does not fully capture [22]. Therefore, while our study provides valuable insights into the impact of RRD on vision-related quality of life, it may not fully reflect the broader implications on patients’ overall well-being. Future studies may consider incorporating general QoL assessments alongside vision-specific tools for a more holistic evaluation. The sample size was relatively small and located at a single institution. Although patients were age-matched, we recognize that statistical adjustment for the matching factor was not carried out in the analysis. This may limit the extent to which potential confounding was fully addressed. Our study did not control for certain known risk factors for RRD, such as high myopia, ocular trauma, and hereditary predisposition. As these factors may independently affect visual function and quality of life, their unequal distribution between cases and controls could have introduced confounding bias. This limitation may affect the internal validity of our findings and should be considered when interpreting the association between RRD and vision-related QoL. Future studies employing stricter matching criteria or multivariate adjustments for these variables are recommended. Our study did not include regression or ROC analyses, nor data visualizations, which could have provided further insights into predictive factors and model performance. We recommend that future studies with larger sample sizes incorporate these methods to enhance generalizability and predictive value. Additionally, A/B-Scan Ultrasound was unavailable; thus, patients with dense ocular media opacity were excluded, which could underestimate the magnitude of RRD in this study. In selecting our control group, we intentionally recruited individuals who were also seeking eye care services, as we aimed to evaluate the impact of RRD on quality of life within a population already engaged with eye health. This approach allowed for a more clinically relevant comparison, highlighting that even among patients attending routine eye check-ups without significant ocular pathology, those with RRD still reported markedly lower vision-related quality of life. In future studies, using stricter control criteria or community-based sampling may help provide a clearer contrast and strengthen external validity. Nonetheless, our current control group still offers relevant insights into comparative visual function outcomes in a real-world clinical setting. The VFQ-25 was not adjusted to accommodate the cultural nuance of the Ghanaian population. For example, the “Driving Difficulty” item is less applicable as many residents of Kumasi, Ghana do not frequently drive themselves. Future studies should address the aforementioned concerns when investigating RRD in low-resource settings. We used the standard NEI VFQ-25 scoring method, which averages subscale scores to generate composite scores. While widely used and useful for comparisons, this method has limitations, including assumptions of equal intervals. Rasch-scaled scoring, suggested by several studies, can offer more precise interval-level measurements and better psychometric validity. However, it requires specialized software and expertise and is not yet standard across VFQ studies. We chose the traditional method for consistency with existing literature, but future studies may benefit from Rasch-based approaches [23]– [24]. It is imperative that attention is focused on expanding retinal services in order to improve overall vision-related quality of life in Ghana. Expanding retinal services should include increasing the quality of retinal examination by non-retinal specialist ophthalmologists, strengthening the referral network, and increasing awareness in the general population about the importance of treating and preventing retinal disease.

While rhegmatogenous retinal detachment (RRD) may not currently represent a top-tier public health priority in Ghana, especially when compared to more prevalent conditions such as cataract or refractive error, our findings still hold important implications for local healthcare delivery. As Ghana’s healthcare system continues to develop, with incremental improvements in infrastructure and specialist training, it is crucial to generate specific data to guide future resource allocation. By highlighting the significant impact of RRD on vision-related quality of life, particularly in the preoperative phase, this study supports the need for greater awareness, early diagnosis, and targeted intervention in facilities where retina specialists are available.

## Conclusion

This study highlights the significantly reduced vision-related quality of life (QoL) among patients with rhegmatogenous retinal detachment (RRD) in Ghana, which emphasizes the critical impact of delayed diagnosis and treatment on visual outcomes. The findings points to the need for improved access to retinal care, increased training opportunities for retinal specialists, and enhanced referral networks in low resource-settings.

## Data Availability

The datasets used and/or analysed during the current study are available from the corresponding author on reasonable request.

## Abbreviations

LMIC: Low and Middle-Income Countries
RRD: Rhegmatogenous Retinal Detachment
NEI-VFQ: National Eye Institute Visual Function Questionnaire
QOL: Quality of Life
